# Circulation of hepatitis B virus genotype-E among outpatients in tertiary hospitals in the Niger-Delta region of Nigeria

**DOI:** 10.4314/ahs.v22i1.60

**Published:** 2022-03

**Authors:** Chinenye F Umego, Clement I Mboto, Atim D Asitok, Linda C Osaji, Uwem E George, Uwem O Edet, Elizabeth N Mbim, Temitope OC Faleye, Olubusuyi M Adewumi, Johnson A Adeniji

**Affiliations:** 1 Department of Microbiology, Faculty of Biological Sciences, University of Calabar, Calabar, Cross River State, Nigeria; 2 Viro-Bio Research Group, Faculty of Biological Sciences, University of Calabar, Calabar, Cross River State, Nigeria; 3 Tansian University Umunya, Anambra State; 4 Breakthrough ACTION, Abuja, Nigeria; 5 Department of Biological Sciences, College of Natural Sciences, Redeemers University, Ede, Osun State, Nigeria; 6 African Centre of Excellence for Genomics of Infectious Diseases, Redeemers University, Ede, Osun State, Nigeria; 7 Department of Microbiology, Faculty of Natural and Applied Sciences, Obong University, Obong Ntak, Etim Ekpo LGA, Akwa Ibom State, Nigeria; 8 Department of Virology, Faculty of Basic Medical Sciences, College of Medicine, University of Ibadan, Ibadan, Nigeria; 9 Center for Human Virology and Genomics, Department of Microbiology, Nigerian Institute of Medical Research, Lagos State, Nigeria; 10 Infectious Disease Institute, College of Medicine, University of Ibadan, Ibadan, Nigeria; 11 WHO National Polio Laboratory, University of Ibadan, Ibadan, Nigeria

**Keywords:** HBsAg, HBV, Niger-Delta, Nigeria, South-South

## Abstract

**Introduction:**

Hepatitis B virus (HBV) infection continues to be a significant public health challenge globally, with higher disease burden in developing countries. HBV genotypes are associated with different geographical regions and clinical outcomes. Limited information exists on epidemiology of HBV in the Niger-Delta region (South-South) of Nigeria. Consequently, this study was designed to characterise hepatitis B virus infection among outpatients in selected tertiary hospitals in the region.

**Methodology:**

Between June and August 2017, consenting nine hundred asymptomatic out-patients were enrolled and initially screened for HBV infection using one step Hepatitis B surface antigen (HBsAg) strip and subsequently re-tested using HBsAg and Hepatitis B core total antibody (anti-HBc) specific Enzyme-Linked Immunosorbent Assay (ELISA). Blood serum with detectable HBsAg were subsequently subjected to DNA extraction, S-gene amplification using a nested polymerase chain reaction (PCR) protocol, gel electrophoresis, sequencing and phylogenetic analysis.

**Results:**

Seroprevalence of HBsAg was 4.6% (95% CI 2.5–7.1) and anti-HBc was 10.1% (95% confidence interval (CI) 6.1–15.3). Of the 41 HBsAg positive samples subjected to DNA extraction and HBV S-gene specific PCR, only 6 (14.6%) yielded the expected ∼408bp band. Phylogenetic analysis based on HBV pre-S/S sequences identified all six typable samples as genotype E, subtype ayw4 of the West African clade.

**Conclusion:**

Results of the study confirm the presence and circulation of HBV genotype-E in the Niger-Delta region of Nigeria, thus corroborating the inclusion of the country in the Genotype E crescent. The authors advocate value-added HBV intervention in the region and the country at large.

## Introduction

Hepatitis B virus (HBV) is a small double-stranded DNA virus and a member of the Hepadnaviridae family^1^. Despite the presence of a prophylactic vaccine with over 400 million people vaccinated, HBV associated hepatitis continues to be a significant public health challenge globally, with more disease burden in the developing countries^2^,^3^. The World Health Organization (WHO) estimates that over 200 million persons are living with HBV infection, with Africa among one of the regions with the highest prevalence of about 60 million (6.1%) infections^4^. Ninety-six per cent of death caused by viral hepatitis were a result of complications of chronic HBV (66%) and HCV (30%) infections^4^. Previous studies conducted in some states in the Niger-Delta (South-South) region of Nigeria have estimated prevalence rates ranging from 4.3% – 8.8%^5^–^11^. However, national surveillance of HBV among apparently healthy population in Nigeria shows a prevalence of 12.2%, with 7.9% of the participants displaying serologic evidence of HBV vaccination-induced immunity^12^.

Hepatitis B virus genotyping is vital in tracing the transmission of the virus, and various geographical regions of the world have reported distinct modes of transmission of HBV^13^,^14^. Specifically, horizontal transmission is common in Africa, whereas, in East Asia, perinatal transmission is more common^15^. Several studies have associated HBV genotype with differences in pathogenicity, disease progression and response to treatment^15^–^19^. For example, it has been observed that genotype E infected patients have a higher frequency of HBeAg positivity and higher viral loads compared to genotype D infected patients^17^. This emphasises the significance of HBV genotyping as a clinical and epidemiological marker^19^. Unfortunately, HBV genotyping is yet to be embraced as a necessary procedure for patient management in Nigeria.

Geographical segregation of HBV genotypes as a result of natural variations in the structure of the surface antigen was first reported by Norder et al.^20^. Subsequently, circulation and predominance of HBV genotype E (HBV/E) in West/Central African crescent spanning from Senegal to Angola was reported^21^–^23^. In Nigeria, HBV genotype E (HBV/E) and HBV genotype A (HBV/A) have been reported in the North-Central and South-Western regions, respectively^13^,^24^–^25^. However, little information exists on the circulating strains of HBV in the Niger-Delta (South-South) region; a tourist hub and crude oil-rich centre of Nigeria. Such information is vital to understanding the evolutionary history of HBV in the country. Consequently, this study was carried out to evaluate HBV infecion among asymptomatic hospital attendees in selected tertiary hospitals in the Niger-Delta region of Nigeria.

## Methodology

### Ethical Statement

Ethical approvals for the study were obtained from the University of Calabar Teaching Hospital (UCTH/HREC/017/JAN/0026), University of Uyo Teaching Hospital (UUTH/AD/S/96/VOL.XXI/528), and University of Port Harcourt Teaching Hospital (UPTH/ADM/90/S.JI/VOL.XI/467) Institutional Review Boards. Participation in the study was voluntary, and each participant provided written informed consent.

### Study area and Design

This study was a hospital-based cross-sectional study conducted in three randomly selected tertiary hospitals located in three different states in the Niger-Delta region of Nigeria. The region is made up of six states covering approximately 25,900 square kilometres. These include Akwa-Ibom, Bayelsa, Cross River, Delta, Edo, and Rivers states. The region is blessed with abundant oil reserve and biological diversity^26^, hence a tourist hub centre.

The study participants were randomly selected from consenting out-patient clinic attendees at the University of Calabar Teaching Hospital (UCTH), Cross River state, University of Uyo Teaching Hospital (UUTH), Akwa Ibom state and University of Port Harcourt Teaching Hospital (UPTH), Rivers State. The selected facilities attend to patients from all over the region^26^. Participants of the study include out-patient, young adults and adults who had lived or worked in the respective areas for at least three years, without prior knowledge of their HBV status and consent to participate in the study. Participants who did not consent to participate in the study were excluded from the study. Enrolment took place between June and August 2017. A total of 900 (300 from each facility) consenting participants aged 17 to 65 years (median age = 31 years) were enrolled for the study.

### Data and Sample collection

Data were collected using an open-ended self-administered questionnaire designed to collect information on socio-demographic characteristics. Subsequently, about five millilitres (5mL) blood was collected into appropriately labelled sterile anticoagulant-free tube from each participant by venipuncture. Blood samples were immediately transported to the Department of Microbiology, University of Calabar in cold chain. Serum was separated from other blood components by low-speed centrifugation at 2,500 rpm for 10 minutes and removed using a sterile disposable pipette. Two aliquots were made per sample into appropriately labelled sterile cryovials and stored away at −20°C until ready for analysis. All sera were later transported in cold chain to the Department of Virology, College of Medicine, University College Hospital, Ibadan for laboratory analysis.

### Laboratory Analysis

#### Hepatitis B Surface Antigen and core total Antibody Detection

All samples were initially tested for HBV infection using one step HBsAg strip (ACON Laboratories incorporated, USA) and subsequently re-tested using HBsAg and antiHBc specific Enzyme-Linked Immunosorbent Assay (ELISA) (MELSIN Diagnostic kits China). Both the one-step HBsAg strip and ELISA assays were performed according to the manufacturer's instructions. For the ELISA assay, optical density (OD) was read using the Emax endpoint ELISA microplate reader (Molecular Devices, California, USA) and the interpretation was made following the manufacturer's instructions.

#### Nucleic acid extraction, HBV partial S-gene amplification and DNA sequencing

Total nucleic acid was isolated from all hepatitis B surface antigen (HBsAg) ELISA positive serum samples (n = 41) using Jena Bioscience Viral RNA+DNA Preparation Kit. The extraction was done following the kit manufacturer's instruction. Subsequently, the HBsAg, specific polymerase chain reaction, was carried out using a nested PCR (nPCR) targeting the partial S-gene region as previously described^13^,^25^. Briefly, both the first and second-round PCR had the same reaction conditions except that DNA extract from the sample was used as the template for first-round PCR while first-round PCR product was used as the template for second-round PCR. Primers HBV_S1 (5-CTAGGACCCCTGCTCGTGTT-3), and HBV_S1R (5-CGAACCACTGAACAAATGGCACT-3), were used for the first-round PCR while HBV_SNF (5-GTTGACAAGAATCCTCACAATACC-3) and HBV_SNR (5-GAGGCCCACTC CCATA-3) were used for the second round. PCR ampification was performed with a total volume of 50µL reaction containing two microliters of each primer (made in 25µM concentrations), 10µL of Red load Taq (Jena Bioscience, Jena, Germany), 4µL of DNA and 32µL of RNase free water. Thermal cycling was done using Veriti Thermal cycler (Applied Biosystems, California, USA.). After denaturation step at 94°C for 3 minutes, PCR reactions were performed in 45 cycles (94°C for 30 seconds, 55°C for 60 seconds and 70°C for 40 seconds with a ramp of 40% from 55°C to 70°C). This was then followed by 72°C for 7 minutes and held at 4°C till terminated. Finally, PCR products were resolved on 2% agarose gels stained with ethidium bromide and viewed using a UV transilluminator. PCR products with required amplicon size were shipped to Macrogen Inc, Seoul, South Korea for PCR product purification and BigDye chemistry sequencing.

### Statistical analysis

Accuracy and completeness of questionnaires were checked, data were double entered to minimise data entry errors and later merged. Data were analysed using SPSS version 21. Descriptive analysis and proportions were calculated, and HBV serological markers prevalence according to the selected sociodemographic factors were calculated using Chi-square, and p-value < 0.05 was considered significant at 95% CI.

### Phylogenetic, mutation analysis and inference of Serotypes

Phylogenetic analysis was performed as previously described^27^, and HBV serotypes were predicted based on analysis of amino acid residues at positions 122, 127, 134 and 160 specifying HBsAg determinants in the S region. Nucleotide sequences of S genes were retrieved from the HBV database (http://hbvdb.ibcp.fr/HBVdb/), and alignment was done using the CLUSTAL W program in MEGA X software with default settings^28^. Thereafter, MEGA X software with the Kimura-2 parameter model^29^ and 1,000 bootstrap replicates was used to construct a neighbour-joining tree.

### DNA sequence submission

The HBV Genotype-E sequences isolated in this study have been deposited at NCBI GenBank under accession numbers MN765091-MN765096.

## Results

### Demographic characteristics and Prevalence of HBV

The age of the 900 out-patients who participated in the study were between 17 and 65 years old, with 39% in the 31–40 age range. There were more female patients (n = 465, 51.6%) than male patients (n = 435, 48.3%). Overall, 91 (10.1%, 95% CI 6.1–15.3) were antiHBc positive and 41(4.6%, 95% CI 2.5–7.1) of these had detectable HBsAg (Table 1). Further analysis showed that participants from UUTH had a higher frequency of antiHBc and HBsAg seropositivity than participants from other locations (7.0%, 13.3% vs 2.3%, 4.0% and 4.3%, 13.0%, p = 0.001). Seroprevalence of HBV infection was higher among participants under 18 years of age (10%, p = 0.034) (Table 1).

**Table 1:** Prevalence of two Hepatitis B virus serological markers by socio-demographic characteristics of HBV study participants

Variable	Test Result for All participants N=900					
	
	HBsAg (-) N (%)	HBsAg (+) N (%)	*p value*	Anti-HBc(-) N (%)	Anti-HBc(+) N (%)	*p value*
**Participants by** **Location**						
UUTH	279(93.0)	21(7.0)	0.023*	260(86.7)	40(13.3)	0.001*
UPTH	293(97.7)	7(2.3)		288(96.0)	12(4.0)	
UCTH	287(95.7)	13(4.3)		261(87.0)	39(13.0)	
**All participants**	859(95.4)	41(4.6)		809(89.9)	91(10.1)	
**Age (years)**						
≤18	45(90.0)	5(10.0)		39(78.0)	11(22.0)	
19–30	195(92.4)	16(7.6)		210(89.0)	26(11.0)	
31 – 40	319(97.6)	8(2.4)	0.031*	322(91.7)	29(8.3)	0.269
41–50	219(95.2)	11(4.8)		209(90.1)	23(9.9)	
≥51	30(96.8)	1(3.2)		29(93.5)	2(6.5)	

**Gender**						
Male	411(94.5)	24(5.5)		388(89.2)	47(10.8)	
Female	447(96.3)	17(3.7)	0.18	420(90.5)	44(9.5)	0.511
**Marital Status**						
Married	481(95.8)	24(4.2)		452(89.5)	53(10.5)	
Single	230(94.3)	14(5.7)	0.27	219(89.8)	25(10.2)	0.926
Divorce	136(97.8)	3(2.2)		126(90.6)	13(9.4)	

**Educational** **Status**						
Primary						
Six/None	251(96.5)	9(3.5)		236(90.7)	24(9.23)	
Secondary	229(95.8)	10(4.2)	0.47	212(88.7)	27(11.3)	0.200
Post-secondary	376(94.5)	22(5.5)		358(89.9)	40(10.1)	

**Occupation**						
Unemployed	253(95.1)	13(4.9)		236(88.7)	30(11.3)	
Self employed	318(94.9)	17(5.1)		297(88.7)	38(11.3)	
Public servant	174(97.2)	5(2.8)	0.52	170(95)	9(5.0)	0.077
Private sector	86(93.5)	6(6.5)		80(87.0)	12(13.0)	

N/B: University of Uyo Teaching Hospital =UUTH, University of Port Harcourt Teaching Hospital =UPTH and University of Calabar Teaching Hospitals =UCTH.

* Statistically significant at p < 0.05

### Phylogenetic and Mutation analysis

Six (14.6%) of the 41 HBsAg positive samples subjected to DNA extraction and HBV S-gene specific PCR, yielded the expected ∼408bp nucleotide sequence band size despite repeated attempts using varied annealing and extension temperatures. All six amplicons were subsequently subjected to sequencing. After combining the forward and reverse sequencing results per isolate into contigs, each was subjected to a BLASTn search on the NCBI BLAST webpage. All the six isolates showed significant similarity to reference HBV S-gene with accession number AB091255 (Table 2). The six isolates were only 7.0% (for NGUUTH_D2) and 6.0% (for the other isolates) divergent (Table 2) from a reference genotype E isolate (AB091255). Using amino acid residues at positions s122, s127, s134 and s160; as earlier described^17^,^25^, all the six isolates were identified to be Genotype E and subtyped as ayw4 (Table 3).

**Table 2:** Comparison of reference sequence gnl|hbvcds|AB091255 genotype E to all isolates recovered from out-patients in selected Tertiary Hospitals in Niger-Delta Region, Nigeria

Species 1	Species 2	Dist %	Similarity %
gnl|hbvcds|AB091255 REFERENCE BACKBONE genotype E	NGUUTH_D2	7.0	98.4
gnl|hbvcds|AB091255 REFERENCE BACKBONE genotype E	NGUCTH_PC03	6.0	98.7
gnl|hbvcds|AB091255 REFERENCE BACKBONE genotype E	NGUCTH_PC01	6.0	98.7
gnl|hbvcds|AB091255 REFERENCE BACKBONE genotype E	NGUCTH_PC06	6.0	98.7
gnl|hbvcds|AB091255 REFERENCE BACKBONE genotype E	NGUUTH_N6	6.0	98.7
gnl|hbvcds|AB091255 REFERENCE BACKBONE genotype E	NGUCTH_PC13	6.0	98.7

**Table 3:** Serotype and Genotype of sequenced HBV isolates recovered from out-patients in selected Tertiary Hospitals in Niger-Delta Region, Nigeria

S/N	Sample ID	Amino acid residues at positions within the surface antigen for serotype determination				Serotype	Genotype
				
		s122	s127	s134	s160		
1	NGUUTH_D2	R	L	F	K	ayw4	E
2	NGUCTH_PC03	R	L	F	K	ayw4	E
3	NGUCTH_PC01	R	L	F	K	ayw4	E
4	NGUCTH_PC06	R	L	F	K	ayw4	E
5	NGUUTH_N6	R	L	F	K	ayw4	E
6	NGUCTH_PC13	R	L	F	K	ayw4	E

**Figure 2:**
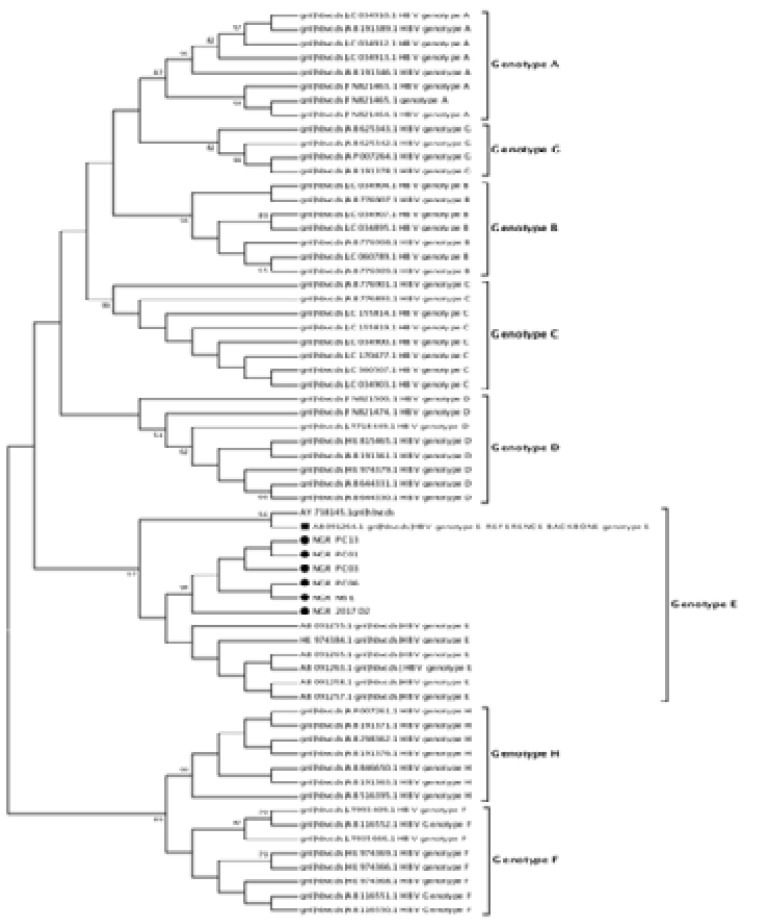
Phylogenetic relationship of recovered HBV Genotypes A-H. The phylogram is based on an alignment of the partial HBsAg sequences. The newly sequenced strains are highlighted with red circle. The evolutionary history was inferred using the Neighbor-Joining method. The optimal tree with the sum of branch length = 0.52352548 is shown. The percentage of replicate trees in which the associated taxa clustered together in the bootstrap test (1000 replicates) are shown next to the branches. The evolutionary distances were computed using the Kimura 2-parameter method and are in the units of the number of base substitutions per site. This analysis involved 67 nucleotide sequences. Codon positions included were 1st+2nd+3rd+Noncoding. All ambiguous positions were removed for each sequence pair (pairwise deletion option). There were total of 444 positions in the final dataset. The GenBank accession numbers of the strains are indicated in the tree. Bootstrap values are indicated if ≥ 50%.

**Figure 3:**
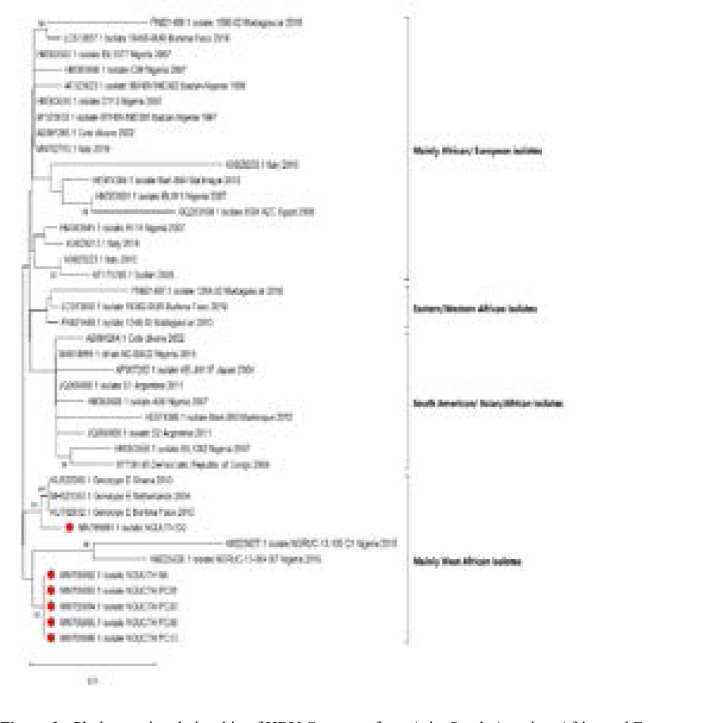
Phylogenetic relationship of HBV Genotype from Asia, South America, Africa and Europe. The evolutionary history was inferred using the Neighbor-Joining method. The optimal tree with the sum of branch length = 0.11425243 is shown. The percentage of replicate trees in which the associated taxa clustered together in the bootstrap test (1000 replicates) are shown next to the branches. The tree is drawn to scale, with branch lengths in the same units as those of the evolutionary distances used to infer the phylogenetic tree. The evolutionary distances were computed using the Kimura 2-parameter method and are in the units of the number of base substitutions per site. This analysis involved 40 nucleotide sequences. All ambiguous positions were removed for each sequence pair (pairwise deletion option). There were total of 441 positions in the final dataset.

In a bid to validate the genotype of the isolates, representative HBV S-gene sequences of genotypes A-H were downloaded from the HBV database (http://hbvdb.ibcp.fr/HBVdb/). These reference sequences alongside HBV S-gene sequences previously described in the region^26^ were used to construct a phylogram. The phylogram also confirmed that all the six isolates previously subtyped as ayw4 are genotype E (Figure 2). Furthermore, the phylogenetic tree of the six isolates with sequences from Asia, Latin America, Europe, and Africa showed that the isolates are of West African origin (Figure 3).

## Discussion

This survey unequivocally reports a moderate prevalence of HBV infection in the Niger Delta region of Nigeria, which is similar to the estimated global prevalence of HBV infection reported by WHO^4^. The observed prevalence rate of 4.6% in this study is classified as moderately endemic based on prevalence rates below 7%^4^. Furthermore, the prevalence of 4.6% observed in this study is lower than the national average of 12.2%^12^, 10% reported by Mbaawuaga et al.^30^ among pregnant women in Makurdi, Nigeria, and 12.3% found among 440 HIV positive patients who were co-positive for HBV^31^. However, the prevalence is higher than the 1.1% recorded among asymptomatic rural community dwellers of Anambra in South-East Nigeria^32^. In Nigeria, most studies on HBV prevalence tend to focus more on high-risk groups such as blood donors and patients with liver disease, which may lead to overestimation of rates. Again, the higher national prevalence estimate may be modelled on higher rates from other parts of the country^33^. Interestingly, we discovered that 10.1% of the participants had anti-HBc antibodies, which indicated that they had been previously infected. Highest HBV prevalence of 7.0% was recorded among out-patients from UUTH (Table 1). The reason for this slightly high prevalence of HBV infection in Uyo is not apparent. However, cultural habits such as unprotected sex, night gathering, including burial night wake keep still practised in Akwa Ibom state could facilitate horizontal transmission of HBV in the population^11^,^15^. The 4.3% prevalence of HBV among out-patients in UCTH in this study is lower than the 5.6% reported during a survey of Chronic liver disease patients in Calabar^9^, and 8.8% prevalence of HBV population screen of HBV in Cross River state^10^. The significant difference in the prevalence rate of this study and other studies done in Cross River state may be as a result of sampling technique, sample population (community-based survey as opposed to hospital-based survey) and sample size. In addition, we observed that HBV seropositivity was highest among participants under 18 years of age and decreased progressively with age, which is at variance with previous report that observed increased progressive HBV seropositivity with age^12^. This may be due to perinatal transmission or infection during early childhood.

Of the 41 HBsAg samples detected in this study, HBV DNA detection assay produced the expected band size in only 6. The inability to detect the expected band size in the remaining thirty-five samples might be due to low viral load in the sample or probably the participants are in the seroconversion phase, or are inactive carriers of HBV. The six isolates from this study showed a divergence of only 6.0–7.0% in S-gene comparison with reference sequence from HBV Carriers in Cote D'Ivoire (AB091255). This observation is similar to previous reports in Nigeria^24^,^25^ as well as Kidd-Ljunggren et al.^34^ who questioned the differentiation of genotype E as a separate monophyletic group distinct from genotype D based on the nucleotide sequences of the X gene.

This study reports the detection of HBV Genotype E in all the six isolates (Table 3). All the six isolates were identified as belonging to subtype ayw4 using amino acids at positions s122, s127, s134 and s160, for isolate classification, thus further confirming the inclusion of Nigeria and Niger-Delta region in particular in the genotype E crescent. Detection of HBV Genotype E in this study agrees with previous findings that HBV/E from Africa^27^,^35^ and Nigeria in particular^13^, ^24^–^25^ belonged to Genotype E serotype ayw4. This is the first report of HBV genotype E serotype ayw4 in Cross River and Akwa Ibom States. Interestingly, the six isolates in this study clustered within the West African clade (figure 3), indicating that this is likely a regional lineage.

**Figure 1:**
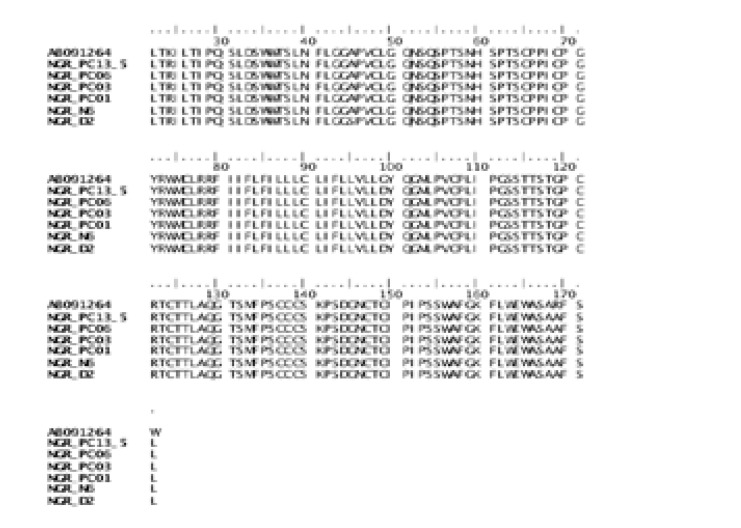
Alignment of amino acid residues of the six isolates sequenced in this study against reference genotype E strains.

HBV has been documented to develop adaptive strategies to overcome host immune mechanisms resulting in the evolution of novel variants. In this case, mutations within the “a” determinant of the major S protein has been described in surface antigen mutants following hepatitis B immunoglobulin treatment or vaccination^36^. Specifically, substitutions at position G145K alongside G145R are associated with immune escape mutants (IEMs)^37^–^39^. In this study, analysis of the variability of the “a” determinant of HBsAg showed that none of the isolates carries mutations previously associated with immune escape mutants (Figure 1). However, we observed a K24R substitution in all the six isolates, and an A45S substitution in isolate NGUUTH_D2 (Figure 1). The effects of these amino acid changes, if any, on evolution and pathogenicity of these viruses are unknown.

## Limitation to this study

The small sample size, and inability to clearly differentiate participants that had current acute infection and those with active or ongoing infection as anti-core-IgM, HBeAg and HBV viral load were not carried out.

## Conclusion

This study describes the moderate prevalence of HBV infection among apparently healthy hospital attendees without prior knowledge of their HBV status in the Niger-Delta (South-South) region of Nigeria. It also confirmed the presence and circulation of HBV Genotype-E in the region, thus corroborating the inclusion of the country in the Genotype E crescent. The authors advocate value-added HBV intervention in the region and the country at large.
